# Can teledentistry be used as an alternative to in-person examinations for the detection of dental caries in pandemic?

**DOI:** 10.1038/s41432-026-01222-2

**Published:** 2026-04-22

**Authors:** Seyyedeh Mahsa Sheikholeslamian

**Affiliations:** Department of Population and Quantitative Health Sciences, School of Medicine, Case Western Reserve University, Cleveland, OH USA

**Keywords:** Health care, Dental diseases

## Abstract

**A Commentary on:**

**Casas K, DiPede L, Toema S, Ogwo C**.

Assessing Teledentistry versus In-Person Examinations to Detect Dental Caries: A Systematic Review and Meta-analysis. *JDR Clinical & Translational Research*. 2025;11(1):4-15. 10.1177/23800844251320974

**Design:**

A systematic review and meta-analysis focusing on the diagnostic accuracy of teledentistry compared to in-person examinations for detecting dental caries in primary and permanent dentition.

The authors conducted a search in PubMed and CINAHL via EBSCOhost and followed the PRISMA-DTA (Preferred Reporting Items for Systematic Reviews and Meta-Analyses for Diagnostic Test Accuracy) guidelines.

**Study selection:**

Comparative studies, including randomized and non-randomized clinical trials, that used DMFT/S or ICDAS indices and reported sensitivity and specificity as diagnostic parameters were considered eligible. Only studies published in English between January 2013 and December 2021 were included. Studies that did not meet these criteria were excluded.

**Data analysis:**

QUADAS-2 and JBI checklists were used for risk of bias and critical appraisal. A DerSimonian–Laird random-effects approach was applied to conduct the univariate meta-analysis. Forest plots were generated for included studies, and sensitivity, specificity, diagnostic odds ratio (DOR), and confidence intervals were calculated. Heterogeneity was assessed using Cochran’s Q and Higgins’s I² to evaluate the variation across studies and the consistency of the results.

**Results:**

Twelve studies reported sensitivity of 45.6–88.3% and specificity of 55.2–98.3% for teledentistry in caries detection. Accuracy ranged from 70% to 96%, and PPV/NPV were 79–92% and 48–97%, respectively. Heterogeneity was low (I² = 24%), and the DerSimonian–Laird random-effects model estimated a diagnostic odds ratio of 35.14, indicating high diagnostic performance.

**Conclusions:**

Teledentistry can be considered a method comparable to in-person examinations for diagnosing dental caries.

## GRADE Rating:



## Commentary

Dental caries is considered one of the most common chronic diseases worldwide.^1^ Routine clinical and radiographic checkups are essential for preventing caries and maintaining oral health. During the COVID-19 pandemic, many people were unable to access dental care, and when they eventually returned, this delay resulted in need on oral and dental services.^2^ In this context, teledentistry has emerged as a helpful tool.^3^ Although virtual consultations are not as ideal as in-person examinations, they provide a practical alternative during situations like a pandemic. In this systematic review and meta-analysis,^4^ the authors aimed to evaluate the diagnostic accuracy of asynchronous and mobile teledentistry modalities compared to in-person examinations for dental caries diagnosis.

After searching the PubMed and CINAHL databases for randomized and non-randomized clinical trials published between January 2013 and December 2021, a total of 12 eligible studies were included in the meta-analysis. The included studies reported diagnostic parameters, including accuracy, specificity, sensitivity, positive predictive value (PPV), negative predictive value (NPV), and kappa statistics to assess using teledentistry among individuals with dental caries. The studies followed PRISMA-DTA guidelines, and each article was assessed using the QUADAS-2 and JBI Critical Appraisal Checklists. Across the studies, teledentistry demonstrated sensitivity ranging from 45.6% to 88.3%, specificity from 55.2% to 98.3%, positive predictive value from 79% to 92%, negative predictive value from 48% to 97%, and overall accuracy from 70% to 96%. These results suggest that teledentistry is more likely to correctly detect caries than to produce false-positive results, with a diagnostic odds ratio of 35.14.

As a strength of this article, the study addresses an important question relevant to the challenges of providing dental care during a pandemic and demonstrates robust statistical analysis. However, there are some minor issues. The reporting of the PRISMA flow shows slight inconsistencies, particularly in the total number of records screened, and the mention of “registers” is unclear, as the methods describe searching only PubMed and CINAHL. This raises questions about whether additional sources, such as trial registries or grey literature, were truly searched, which could affect the comprehensiveness of the review. Some reasons reported for excluding articles at the full-text screening stage, such as “Does not assess caries” and “Teledentistry is used for oral health education” seem more relevant as an exclusion criteria for title and abstract screening. The authors also did not describe the screening process in detail, such as whether any software was used or how consistency among reviewers was ensured, for example through pilot screening. Additionally, although the meta-analysis provides useful pooled estimates, most of the included studies are observational or retrospective, and not all represent true head-to-head comparisons of teledentistry versus in-person clinical examinations. The differences between examiners, study designs and the inclusion of pre and post COVID studies may limit the generalizability of the results, even though heterogeneity was reported to be low. While the meta-analysis offers valuable insights, the authors did not use a formal framework such as GRADE^5^ to evaluate the certainty of evidence, which could have provided clinicians with a clearer understanding of the confidence in these findings.

From a clinical perspective, in-person examination combined with radiographic imaging remains essential for accurate caries diagnosis. While the high diagnostic odds ratio reported in this SRMA suggests that teledentistry can reliably identify caries, it cannot fully replace direct clinical evaluation, including radiographs. The wide range of sensitivity observed in the included studies is a concern, as lower sensitivity indicates that some caries cases may be missed during remote assessment. In situations such as the COVID-19 pandemic or other urgent circumstances, patients may delay or miss follow-up visits, which could lead to progression of untreated caries and more serious dental complications. Therefore, teledentistry should be considered primarily as a screening or triage tool to identify patients who require timely in-person care rather than a standalone diagnostic method. Future research, ideally randomized trials with standardized methodologies and modern imaging technologies, will be essential to further strengthen the evidence base and guide clinical implementation.

Practice Points

- Teledentistry assessments, as a remote evaluation tool, can detect and diagnose dental caries and can be considered an option when in-person visits with a dentist are not possible, such as during a pandemic.
- To provide stronger clinical guidance, future studies should include well-designed randomized controlled trials to confirm the diagnostic accuracy of teledentistry compared with in-person examinations
